# Physicochemical Characterization of Mining Waste from the Betare-Oya Gold Area (East Cameroon) and an Adsorption Test by Sabga Smectite (North-West Cameroon)

**DOI:** 10.1155/2020/6293819

**Published:** 2020-10-09

**Authors:** Mambou Ngueyep Luc Leroy, Mache Jacques Richard, Ayiwouo Ngounouno Mouhamed, Takougang Kingni Sifeu, Abende Sayom Reynolds Yvan, Roukaiyatou Said

**Affiliations:** ^1^Department of Mining Engineering, School of Geology and Mining Engineering, University of Ngaoundéré, P.O. Box 115, Meiganga, Cameroon; ^2^Department of Mechanical Petroleum and Gas Engineering, Faculty of Mines and Petroleum Industries, University of Maroua, P.O. Box 46, Maroua, Cameroon

## Abstract

This paper, firstly, characterizes the mining waste of the Betare-Oya gold area (East Cameroon). Surface waters are sampled and characterized physically (pH, electrical conductivity, turbidity, and suspended solids) and chemically by the determination of trace metals (Cu, Zn, Ni, Mn, Fe, Cr, As, and Pb). The concentrations of heavy metals in sediment samples are determined by X-ray Fluorescence Spectrometry (XRF) analysis. The statistical analysis tool is used to determine the existing correlation between the different physicochemical parameters. Secondly, an adsorption test for heavy metals determined is carried out on smectic clay of Sabga (North-West Cameroon) at ambient temperature. The results of physicochemical characterization of water samples reveal that waters of this gold mining zone are slightly acidic to neutral (6.3 pH < 7.1), mineralized (18.54 EC < 43 *μ*s.cm^−1^), turbid (60 SS < 237.67 NTU), and polluted by suspended solids (50.5 < SS < 666.6 mg L^−1^).The determination of heavy metals in water samples shows that manganese, iron, arsenic, and lead concentrations are above the World Health Organization (WHO) limits. This indicates pollution. High concentrations of heavy metals as Cu, Ni, Mn, Fe, and Cr are observed in sediment samples. The correlation analysis indicates high correlations between turbidity and suspended solids; cadmium and lead in water samples; and copper and iron in sediment samples. The adsorption test reveals that the maximum percentage removal is 60, 89, 55, −89.74, and 100% for Cu, Zn, Mn, Fe, and Pb, respectively, after adding 0.5 g of Sabga smectic clay. This clay has a good potential adsorption of lead, zinc, manganese, and copper. However, the operating conditions favored desorption of iron.

## 1. Introduction

Water is at the center of all socioeconomic processes, regardless of the level of development of society. Increasing mining activities are putting increasing pressure on the planet's fresh water supplies. Indeed, these activities generate a wide variety of chemicals which flow into the water cycle, endangering the fragile natural balance which has allowed life to develop on Earth. Often, the chemicals in wastewater are difficult to biodegrade, and the lack or inadequacy of treatment systems leads to their accumulation in the water cycle [1].

Extraction of solid minerals has been identified as a major entry point for heavy metals into the environment, thereby polluting various environmental components such as soil, water, and air [2]. In the process of extracting particular metal, the entire mass of soil is excavated and exposed to environmental agents of weathering, degradation, and transport, which leads to soil erosion and significant contamination of surrounding areas. As in most developing countries, Cameroon has seen great progress in gold mining, mainly in the Adamawa and Eastern regions (Batouri, Colomines, Yokadouma, Meiganga, and Betare-Oya [3]). In the latter case, the gold sites are exploited in an artisanal to semimechanized manner, thus predisposing surface waters to serious environmental pollution [4]. The mining perimeter of Betare-Oya chosen as the study area is the victim of artisanal and semimechanized anarchic mining operations and subject to such pollution risks. Solid, liquid, physical, and chemical waste resulting from artisanal activities and the semimechanized exploitation of gold deposits in the region could lead to the degradation of the water quality of the region and even of the Sanaga whose Lom is a tributary.

Worldwide, several studies have been conducted on the impacts of industrial, artisanal, and semimechanized mining on water resources and their contamination by heavy metals [5–9]. These works support the fact that the release of heavy metals in surface water is mainly due to the alteration of tailings and waste rock. In Cameroon, very few studies have been carried out on the physicochemical characterization of the waters and sediments of a mining operation. Some authors [3, 10, 11] studied the impacts of gold mining exploitation on the surface water quality.

Some techniques have been used to eliminate pollutants in waste waters. Polyaluminum silicate chloride (PASiC) and the electrocoagulation (EC) process have been used to remove organic matter in surface water [12]. Nitrate was eliminated in aqueous solutions by using NF membranes under different circumstances (nanofiltration) [13]. The photocatalytic ozonation process was used to degrade ciprofloxacin pollutant with ZnO nanoparticles [14]. Malakootian et al. [15] investigated the efficiency of the microbial desalination cell in the removal of arsenic from aqueous solutions. Recently, the ozonation process with calcium peroxide was used to remove ciprofloxacin [16] and heavy metals as Pb, Cu, Zn, Ni, and Cd [17]. Compared to the adsorption process, these techniques are more expensive. Research in the fields of adsorption for the treatment of polluted water is of global scope. Many adsorbents have been used to remove pollutants and, specifically, heavy metals in waters. Malakootian et al. [18] presented a review, which summarized the most relevant works about the use of different nanoparticles to remove antibiotics from aqueous solutions. Specifically, in [19, 20], nanoparticles were used to remove bisphenol and nonylphenol from aqueous solutions. Sharifpour [21] investigated the usage of single-walled carbon nanotubes for the removal of penicillin G from aqueous environments. CoFe_2_O_4_-activated carbon chitosan prepared as a new magnetic nanobiocomposite was used for adsorption of ciprofloxacin (CIP) [22]. Red soils and activated bentonite were used to eliminate Cu (II) and Zn (II) from acid copper mine drainage [23]. The choice of the optimal adsorbent in terms of adsorption capacity and operating costs is a perpetual quest by researchers, hence the tendency to fall back on more available and abundant natural adsorbents. Research has been carried out on adsorption using clay minerals to remove various pollutants (heavy metals, cationic dyes, acid dyes, herbicides, and hydrocarbons) on water polluted [24–26]. Adsorption, therefore, appears to be an appropriate technique. In Cameroon, several researchers have studied the adsorption of heavy metals on clays from different localities [27, 28]. Sabga smectite has been used for the retention of Cd (II) and Co (II) ions [29, 30] and also amaranth [31].

The main objective of this study is to characterize, physically and chemically, surface water and sediments in the gold mining environment of the study area in Betare-Oya (East Cameroon) and to carry out an elimination test by adsorption of heavy metals on the smectic clay of Sabga (North-West Cameroon). This work is organized as follows: the second section presents materials and methods. The third section presents results and discussions. The last section gives the conclusion which recalls the original results and the main contributions of this study.

## 2. Materials and Methods

**Figure 1:**
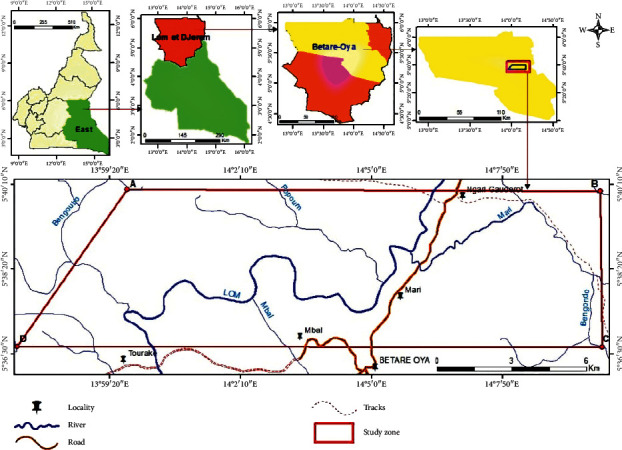
Location map of the study area.

The location map of the study area is presented in Figure 1.

The Betare-Oya gold district is located in the eastern region of Cameroon, Lom and Djerem department. It extends in the Sanaga basin between latitudes 5°30′07″ and 5°F′01″ North and longitudes 14°04′04″ and 14°28′06″ East (Figure 1). The study area is located in the Laï canton, precisely in the Mari village at 5 km north of Betare-Oya. The climate is equatorial type with four seasons (2 dry seasons and 2 rainy seasons) with some variations which are particular to them due to the location at the foot of the Adamawa Plateau. The Betare-Oya gold districts are watered by two main rivers: The Lom and the Pangar. These two rivers receive water from a large network of small rivers, the most important of which are Mba, Mari, Mbal, and Kpawara. The hydrologic regime of the river Lom in Betare-Oya is controlled by rainfall. The lower monthly flow rate is observed in February (56 m^3^ s^−1^), while the maximum flow rate is observed in October (328 m^3^ s^−1^).

### 2.1. Sampling, Physical, and Chemical Parameters of Waters and Sediments

**Figure 2:**
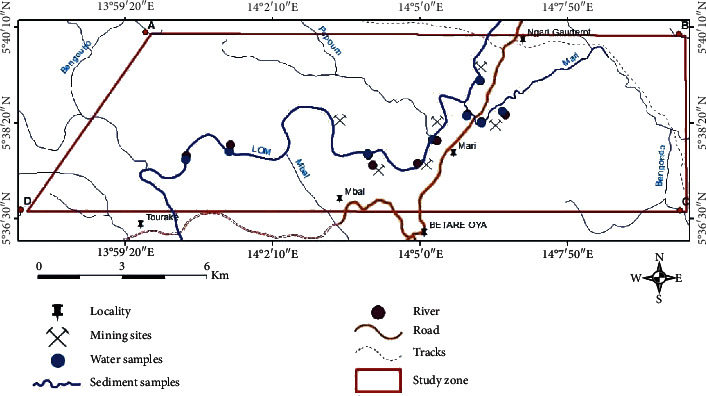
Sampling map.

For this study, eight water samples (WS1, WS2, WS3, WS4, WS5, WS6, WS7 and WS8) are taken from the surface waters of artisanal and semimechanized gold mining on the Mari site (Betare-Oya). The samples were taken between 9 a.m. and 1 p.m on July 12, 2017. The water samples are taken using plastic bottles rinsed thoroughly with the water sampled and, then, introduced into the water to a depth of 30 cm. Until completely filled, closed, then acidified, and stored directly in a cooler. Ten sediment samples (SS1, SS2, SS3, SS4, SS5, SS6, SS7, SS8, SS9, and SS10) are also taken from the operating sites near the various sampling points for the water samples and, then, placed in plastic bags and kept in the cooler. The sampling map is presented in Figure 2.

The geographical coordinates of water and sediment samples are summarized in Table 1.

To characterize, physically, the water samples taken from the gold district, many parameters are determined. These parameters are pH, electrical conductivity (EC), turbidity (Tu), and suspended solids (SS). The pH analysis is carried out in the chemistry laboratory, School of Geology and Mining Engineering of the University of Ngaoundere, Cameroon. Electrical conductivity, turbidity, and suspended solids are measured using the analytical protocols of the Chemical Engineering Laboratory of the University Institute of Technology of the University of Ngaoundere, Cameroon. The analysis of heavy metals is carried out at the International Institute of Tropical Agriculture Laboratory of Analysis and the Pastor Center of Yaounde by atomic absorption spectrometry (AAS). AAS is a spectroscopy technique used to determine the concentration of trace metals (alkali metals, alkaline earth metals, and transition metals), as well as metalloids, in a sample. The analyzed metals are copper, zinc, nickel, manganese, chromium, arsenic, lead, and iron. Ten sediment samples are analyzed by using a portable XRF (Gemius 7000) to determine metal and trace element concentrations at the laboratory of School of Geology and Mining Engineering of the University of Ngaoundere.

### 2.2. Correlation Matrix

The correlation matrix used in the work is Pearson's correlation [32].

(1) $$\begin{array}{c} r=\;\frac{\sum^{}\left(X-\bar{X}\right)\left(Y-\bar{Y}\right)}{\sqrt{\sum^{}{\left(X-\bar{X}\right)}^{2}\sqrt{\sum^{}{\left(Y-\bar{Y}\right)}^{2}}}}, \end{array}$$

where $\bar{X}$  = mean of *X* variable and $\bar{Y}$  = mean of *Y* variable.

### 2.3. Characterization of the Smectite and Its Adsorption Properties

The material used is the smectic clay of Sabga (North-West Cameroon) denoted as SB01. The characterization of this clay is limited only to physical and textural properties. The cation exchange capacities (CEC) are measured by saturating 300 mg of the fraction <250 *μ*m using ammonium acetate (1 N, pH = 7).The pH of the natural samples is measured using a TIM 845 analytical radiometer after stirring the water suspensions at 1 : 5/milli Q for 10 minutes and for 30 minutes. The specific surfaces and the total pore volume of the samples are determined from nitrogen adsorption and desorption isotherms at 77 K obtained from a Carlo Erba Sorptomatic 1990 volumetric device after degassing the samples overnight at room temperature under a pressure below 10–4 Pa. The specific surface area (SSA) is calculated from the adsorption data by applying the method of Brunauer–Emmet–Teller (B.E.T). These parameters are measured following the laboratory protocol of the Research Unit Clays, Geochemistry and Sediment Environment of the University of Liege.

Chemical analyses are carried out by emission spectrometry at Nancy, France. Approximately 1 g of clay < 250 *μ*m powder was molded in fused lithium borate (LiBO_2_) and dissolved in nitric acid. Inductive Coupled Plasma by Atomic Emission Spectrometry (ICP-AES) is used for the determination of the major elements, and Inductive Coupled Plasma by Mass Spectrometry (ICP-MS) is used for trace elements. Relative analytical uncertainties were estimated at 1–5% for major elements except for P_2_O_5_ (10%). They are up to 5% for most of the trace element concentrations except for Cu (10%). However, uncertainty was high (>10%) for any trace element displaying a low concentration (<0.1 ppm).

The adsorption test is studied as a function of the adsorbent mass. The experiments are performed at ambient temperature. In a 125 mL beaker, 0.5 g and, then, 1 g of smectic clay are brought into contact with 50 mL of water (WS3) containing copper, zinc, manganese, iron, and lead. The sample is agitated for 1 hour using an IKA RT 5 magnetic stirrer at a temperature of 25°C and a speed of 800 rpm. The adsorption efficiency (%) is calculated according to the expressions:

(2) $$\begin{array}{c} \%\text{ Adsorption}=\;\frac{\left(C_{0}-\;C_{f}\right)}{C_{0}}\;\times 100, \end{array}$$

where *C*_0_ is the initial concentration (mg·L^−1^) and *C*_*f*_ is the solution concentration at the end of the sorption process (mg·L^−1^) [33]. The adsorption capacities at equilibrium, *q*_*e*_ (mg·g^−1^), of the metals are calculated using the following equation:

(3) $$\begin{array}{c} q_{\max}=\;\left(C_{0}-\;C_{e}\right)\cdot \frac{V}{W}, \end{array}$$

where *C*_0 _ and *C*_*e*_ (mg·L^−1^) are the liquid-phase concentrations of heavy metals at time zero and equilibrium, respectively. The parameter *V* is the volume (mL) of the sample solution and the parameter *W* is the weight (mg) of the dry sorbent.

## 3. Results and Discussions

### 3.1. Physical Parameters

**Figure 3:**
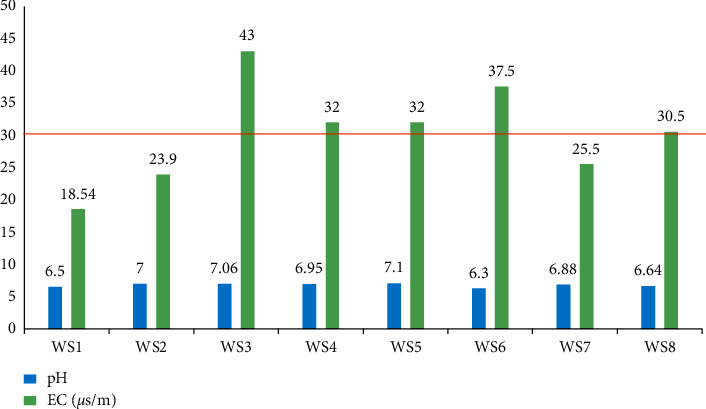
Physical characteristics (pH and EC) of the sampling waters.

**Figure 4:**
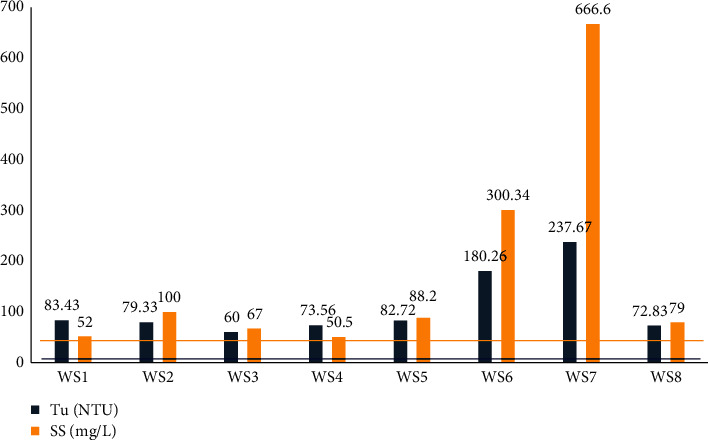
Physical characteristics (Tu and SS) of the sampling waters.

Physical analyses results of water samples are presented in Figure 3 (pH and electrical conductivity) and in Figure 4 (turbidity and suspended solids).

The pH values of the waters studied are slightly acidic to neutral and range from 6.3 to 7.1, as shown in Figure 3. These values are not in agreement with the range of values recommended by the WHO standard (6.5–8.5) [34] for the WS6 sample. The pH values of the different samples express a slight acidity, but still remain close to one of the other. This slight acidity is attributed to the presence of sulfides, in particular the pyrite accompanying the gold, the hydrolysis of which acidifies the environment. Such pH ranges systematically induce an increase in the rate of absorption of trace elements in surface sediments. Electrical conductivity (EC) is probably one of the simplest and most important properties for controlling the quality of water. It reflects the degree of overall mineralization and provides us with information on the salinity rate. The conductivity values are above the standard value of WHO (see Figure 3) for all the samples. This indicates that waters are mineralized [35]. This could be due, on the one hand, to the time of contact with sediments and soils which is much longer and, on the other hand, to the exposure of mine tailings, so leaching is more pronounced.

The turbidity of a water is due to the presence of finely divided suspended matter: clay, silts, silica grains, and organic matter. Turbidity values of the water samples taken vary between 60 NTU and 237.67 NTU, as shown in Figure 4. These values greatly exceed the limit of the WHO standard for drinking water set at 5 NTU [34]. From the point of view of turbidity, this pollution comes from the contribution of fine particles of organic and mineral nature contained in the soils and in the washing discharges. The variations in turbidity are substantially proportional to those of the SS. The SS values are above the WHO standard (50 mg L^−1^) at all the sampling points. The WS7 sample has a high value compared to the other samples because it is placed at the exit of the large site operation. A comparison with the values found by Nicolau et al. and Olivry [36, 37] in Ntem and Nyong, respectively (6 mg L^−1^ ≤ SS ≤ 30 mg L^−1^), shows that the SS values in the samples are higher. Therefore, these great values are attributed to artisanal and semimechanized exploitation which, through the various activities carried out such as deforestation, digging in the river bed, or even the exposure of washing discharges, contributes to their increase.

### 3.2. Heavy Metals in Water Samples

**Figure 5:**
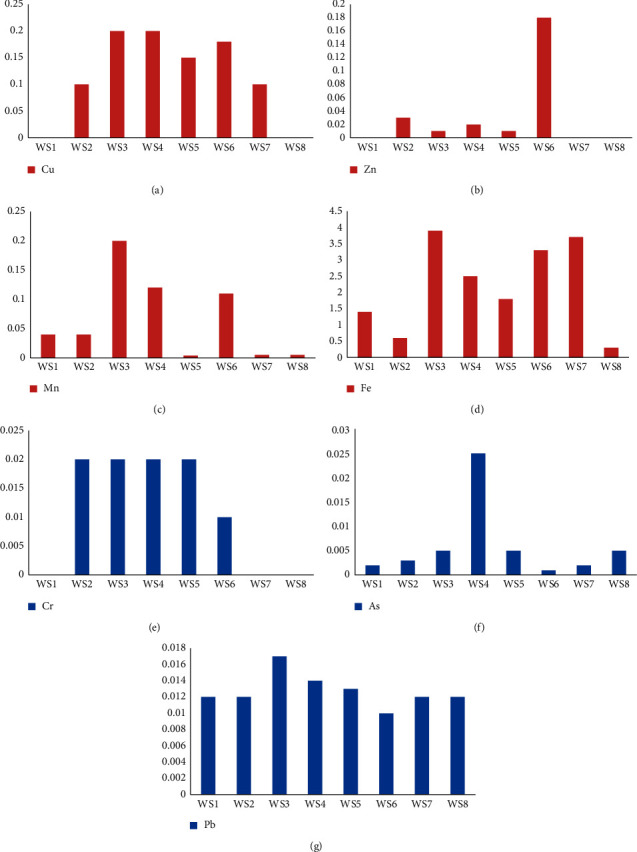
Spatial variations of heavy metals analyzed in water samples: (a) copper; (b) zinc; (c) manganese; (d) iron; (e) chromium; (f) arsenic; and (g) lead.

The relative concentration of heavy metals is presented in Table 2, and the spatial variations of heavy metals analyzed in water samples are illustrated in Figure 5.

In Figure 5, the concentrations of copper at sampling points are below the WHO standard (2 mg L^−1^). The presence of copper in water samples may also be due to soil leaching and the weathering of the chacopyrite.

Zinc concentrations are below the WHO standard, which indicated a slight zinc contamination of the study area. The sphalerite mineral is the main source of zinc. The dissolution of this mineral can contaminate surface water. Zinc concentrations are not elevated in gold zones [3, 9].

For all the water samples, manganese concentrations are between 0.04 and 0.2 mg L^−1^ (see Figure 5(c)). This value is much lower than the WHO standard (0.05 mg L^−1^). Manganese is one of the most abundant metals after iron [38]. Although the sediments are rich in manganese, its remobilization in water is limited by the pH range of the different water samples greater than 6. The affinity of manganese for the solid phase decreases with pH [39].

The iron concentrations of our samples vary from 0.34 mg L^−1^ to 3.9 mg L^−1^ (Figure 5(b)). These values are above the WHO standard (0.3 mg L^−1^). Iron is the most abundant element in the various water samples, as well as in the sediments and the geochemical bottom. Therefore, it has greater bioavailability. The high iron contents in the waters are, firstly, due to the leaching of the lateritic grounds which confirms the results of [40]. Secondly, this is due to the hydrolysis of the pyrite which releases iron. Higher concentrations of iron are also observed in other gold zones [10, 11].

Chromium concentrations are equal to 0.02 mg. L^−1^ at all sampling points. These results are very far from the maximum admissible value set by WHO at 0.05 mg. L^−1^. The chromium is contained in most soils and rocks.

Arsenic levels are above the WHO standard at certain points. The presence of arsenic in the waters of the study area could be due to leaching of the soil. The soils contain arsenic primary minerals resulting directly from the alteration of the bedrock in the subsoil where the most frequent species of primary mineralization are not only arsenopyrite (FeAsS), realgar (AsS), and orpiment (As_2_S_3_) but also niccolite (NiAs), cobaltite (CoAsS), tennantite (Cu_12_As_4_S_13_), enargite (Cu_3_AsS_4_), proustite (Ag_3_AsS_3_), and native arsenic. The lead concentrations are above the WHO standard at all sampling points. This indicates the pollution. They are also higher than lead levels in the two gold zones of Betare-Oya and Hire [3, 9].

The lead concentrations are above the WHO limit in the WS4 sample. The main source of lead in nature is galena (PbS). The presence of lead in water samples mainly can also be due to the leaching of discharges from the farm. Lead fuels from mining machinery are a secondary source. In aquatic systems, lead has a strong affinity for sediment particles, including clays, iron, and manganese Oxyhydroxides, sulfides, and organic matter [41].

### 3.3. Heavy Metals in Sediments

XRF analysis carried out enabled to have the heavy metal concentrations studied for the sediment samples taken from the study area. The results are presented in Table 3.

In Table 3, the concentration of copper is more than the limits of the WHO standard (30 ppm) for SS3, SS4, SS5, SS9, and SS10. This implies that the sediments are polluted with copper. The highest copper can be due to the fact that retention would be governed by ion exchange with clays such as the kaolinite and by complexing with fulvic acids and humic acids [42, 43]. The nickel concentrations are also above the WHO standard at certain sampling points (SS9 and SS10). It can be due to the batteries and iron and steel equipment used. Zinc concentrations are below the WHO standard at all the sampling points. The concentrations of manganese and iron are very high in all the sediment samples. For iron, this can be due to the presence of pyrite and also the influence of the mining activity. Two types of soils are present in this area, particularly ferruginous tropical soils (the majority) and ferralitic soils. The concentrations of chromium are above the WHO standard for SS3, SS4, SS7, SS8, and SS10 samples. High concentrations of chromium in soils are an environmental problem because of its high toxicity. The presence of chromium can be due to the oxidation of ultramafic rocks or the leaching of ultramafic soils which is large and controlled by the amorphous Fe-oxides [44]. Arsenic concentrations are below the WHO standard. For SS8, the concentration is substantially equal to the WHO standard. Arsenic is a frequent element in subsurface soils because it is coprecipitated with sulfides or sorbed with iron oxyhydroxides.

### 3.4. Correlation Matrix

The correlation matrix of parameters determined in water and sediment samples is presented in Tables 4 and 5.

In Table 4, correlations are obtained between parameters, such as pH and EC (0.145) and pH with all heavy metals except Zn (−0.693). Electrical conductivity has a high correlation with Cu (0.732) and Mn (0.714). Turbidity has high correlations with SS (0.965). This correlation is attributed to physical pollution, which probably originated from the erosion of mining wastes (tailings) and discharge of wastewater from gold washing into the rivers. Secondly, for the heavy metals, the matrix correlation obtains indicated that the Cu/Cr (0.765), Mn/Fe (0.586), Cd/Pb (0.89), and Cr/Pb (0.509). Generally, the tailings from gold extraction are a major source of heavy metals in water, and so are the chemical products used during the separation of gold and operation of excavating machines (excavators and heavy-duty trucks). However, these elements can sometimes be derived from geological units, for example, Cd exists in spharelite (ZnS) accompanied by Zn; iron and arsenic are linked to the oxidation of sulfide minerals (pyrite FeS2 and arsenopyrite FeAsS) or rocks containing gold bearing ores [45]. Considering the high concentration of some elements, their origins can be attributed to the contributions of anthropogenic factors.

From Table 5, a high correlation can be seen between the Cu and Fe (0.773). Ni has a correlation with Pb (0.482) and Cr with As (0.660). The origin can be from mining tailings deposited along the Lom River. The significant correlation observed between these parameters suggests that the origin can be the mining activity or from geological formations; also, others are from agricultural processes.

### 3.5. Adsorption Test by Using Sabga Smectic Clay

The physical characteristics of Sabga smectic clay are presented in Table 6.

In Table 6, the sample has an acid equilibrium pH. The cobaliti hexane method revealed the CEC value of 42 meq/100 g. The specific surface area of the sample is 90 m^2^ g^−1^. This high specific surface area confirms the high smectite mineral content in this clay [28]. It is an important factor when the clay is used as an adsorbent. The composition in oxides of Sabga clay is presented in Table 7.

Table 7 shows the composition in the oxides of the Sabga clay sample. The most abundant oxides are SiO_2_, Al_2_O_3_, and Fe_2_O_3_ whereas K_2_O, CaO, Na_2_O, MgO, TiO_2_, and MnO are present only in small quantities. The SiO_2_ value is 63.23%. The Al_2_O_3_ content is 13.57%. The SiO_2_/Al_2_O_3_ ratio is greater than 4 in this sample (5.03) owing to the sandy content of the sample. The loss on ignition of the sample has ranged from 10.51%. The mineral composition of Sabga clay is presented in Table 8.

From Table 8, it can be seen that montmorillonite is the mineral present in the highest abundance (minimum 44%), followed by cristobalite and feldspar. The WS3 sample is used to study the adsorption of heavy metals on Sabga clay at ambient temperature. Sample WS3 is used because it contains higher heavy metal concentrations. The results of the adsorption test by using smectic clay are shown in Tables 9 and 10.

In Table 9, the maximum percentage removal is 60, 89, 55, −89.74, and 100% and the adsorption capacities are 0.6, 17.8, 2.2, negative, and 1.1 mg g^−1^ for Cu, Zn, Mn, Fe, and Pb, respectively. Lead is favorable to adsorption by Sabga smectite at neutral pH.

The results of the adsorption test on the filtrates show that Sabga clay interacts with water. The maximum percentage removal is 80, 83.5, 45, −140.51, and 100% and the adsorption capacities are 0.4, 8.35, 0.9, and 0.55 mg g^−1^for Cu, Zn, Mn, Fe, and Pb, respectively. The maximum percentage of adsorption of lead (II) is higher than that observed in [46] with wood ash in the same pH. Sabga smectite is, therefore, suitable for the elimination of copper, zinc, manganese, and lead at ambient temperature and at a pH of 6.88. However, the opposite happened for Iron which is desorbed from the clay. The desorption can be due to the fact that the available sites on the clay are not favorable to the binding of iron to the adsorption conditions used. Secondly, the iron contained in the clay is released during the adsorption process. Indeed, for some metals, it is necessary to go to higher temperatures and lower pH. The obtained results show that Sabga smectite, as an available and low cost matter, can be considered as an alternative in the field of heavy metals removal from this type of wastewater and similar wastewater. The adsorption capacity values of the Sabga smectite for manganese and lead are compared to other adsorbents in Tables 11 and 12.

The results of this study are compared with other similar works in Table 13.

## 4. Conclusions

The main objective of this paper was to carry out the physicochemical characterization of surface waters and sediments of the study area located in the Betare-Oya gold district (East, Cameroon). Secondly, this paper was presented in an attempt to remove heavy metals by using Sabga smectite (North-West Cameroon). The results of the characterization showed that the surface waters are slightly acidic to neutral, very weakly mineralized, very turbid, and polluted by suspended solids. Heavy metals analyses of water samples revealed that zinc, nickel, chromium, and copper are below the thresholds defined by the World Health Organization standards. Heavy metals such as manganese, arsenic, and lead andiron, particularly, were attracting attention because their concentrations that are above the thresholds admissible by the World Health Organization standards. Indeed, deforesting operations, storage of mine wastes, and the creation of excavations make available the various minerals and metals present in soils and mine wastes and facilitate their leaching and transport. The presence of these metals can be also be due to the weathering of minerals and rocks present in the study area. The heavy metals were also determined in the sediment samples by X-ray Fluorescence Spectrometry analysis. They were observed a high predominance of iron and manganese concentrations. The sediments were polluted by copper, nickel and chrome. According to the adsorption test, the maximum percentage removal of 60, 89, 55, −89.74, and 100% for Cu, Zn, Mn, Fe, and Pb, respectively, was observed. The heavy metals removal test by adsorption on Sabga smectite showed a net effectiveness for the elimination of lead, manganese, copper, and zinc and the opposite effect on iron, which was desorbed because the potential hydrogen and temperature were not ideal for their adsorption. Sabga smectite was an available and low-cost matter and can be considered as an alternative in the field of heavy metals removal from this type of wastewater and similar wastewater.

## Figures and Tables

**Table 1:** Geographical coordinates of samples.

Samples		Coordinates	
		Latitude	Longitude
WS1	SS1	5.641510	14.110580
WS2	SS2	5.652193	14.102494
WS3	SS3	5.639261	14.102823
WS4	SS4	5.641627	14.098377
WS5	SS5	5.633145	14.088783
WS6	SS6	5.628493	14.066783
WS7	SS7	5.631660	14.022550
WS8	SS8	5.628210	14.008448
	SS9	5.621025	14.068178
	SS10	5.625853	14.082521

**Table 2:** Relative concentration of the heavy metals analyzed.

Parameters	Unit	Concentrations			WHO standards
		Min	Med	Max	
Cu	mg·L^−1^	0	0.005	0.01	2
Zn	mg·L^−1^	0	0.015	0.03	3
Ni	mg·L^−1^	0	0	0	0.02
Mn	mg·L^−1^	0.04	0.12	0.2	0.05
Fe	mg·L^−1^	0.4	2.15	3.9	0.3
Cr	mg·L^−1^	0.02	0.02	0.02	0.05
As	mg·L^−1^	0.005	0.015	0.025	0.01
Pb	mg·L^−1^	0.012	0.018	0.024	0.01

**Table 3:** Concentrations of heavy metals in sediment samples.

Samples	Unit	Cu	Ni	Zn	Mn	Fe	Cr	As	Pb
SS1	ppm	4.92	34.6	22.87	676.45	39360.38	66.9	3.5	32
SS2	ppm	0	42.3	10.09	201.26	12390.46	102.4	5.98	26.86
SS3	ppm	115.8	31.8	15.5	894.42	70110.35	79.93	8.57	27.09
SS4	ppm	84.83	53.54	23.61	847.51	56203.97	105.84	5.68	18.52
SS5	ppm	48.42	8.56	12.4	1076.4	45403.65	52.85	5.4	16.36
SS6	ppm	15.01	10.34	17	636.73	42914.73	16.51	2.87	8.06
SS7	ppm	23.84	7.67	11.88	744.79	49391.44	119.92	8.05	13.54
SS8	ppm	11.85	50.4	16.01	430.51	41723.8	94.73	9.34	11.56
SS9	ppm	58.01	63.68	18.06	325.56	70305.35	60.78	3.45	23.68
SS10	ppm	83.76	61.52	21.77	436.35	52395.13	100.56	5.7	29
WHO	ppm	30	50	90	—	—	70	10	35

**Table 4:** Correlation matrix between parameters determined in water samples.

Variables	pH	EC	Tu	SS	Cu	Zn	Mn	Fe	Cr	As	Pb
pH	**1**	0.145	−0.335	−0.135	0.370	−0.693	0.068	0.057	0.638	0.328	0.689
EC	0.145	**1**	−0.100	−0.110	**0.732**	0.373	**0.714**	0.551	0.483	0.158	0.483
Tu	−0.335	−0.100	**1**	**0.965**	0.059	0.445	−0.248	0.522	−0.465	−0.353	-0.524
SS	−0.135	−0.110	**0.965**	**1**	0.035	0.229	−0.274	0.524	−0.448	−0.336	-0.366
Cu	0.370	**0.732**	0.059	0.035	**1**	0.323	0.687	0.681	**0.765**	0.412	0.419
Zn	−0.693	0.373	0.445	0.229	0.323	**1**	0.257	0.322	−0.037	-0.251	−0.539
Mn	0.068	**0.714**	−0.248	−0.274	0.687	0.257	**1**	0.586	0.503	0.313	0.617
Fe	0.057	0.551	0.522	0.524	0.681	0.322	0.586	**1**	0.126	0.037	0.331
Cr	0.638	0.483	−0.465	−0.448	**0.765**	−0.037	0.503	0.126	**1**	0.423	0.509
As	0.328	0.158	−0.353	−0.336	0.412	−0.251	0.313	0.037	0.423	**1**	0.373
Pb	0.689	0.483	−0.524	−0.366	0.419	−0.539	0.617	0.331	0.509	0.373	**1**

**Table 5:** Correlation matrix between heavy metals determined in sediment samples.

Variables	Cu	Ni	Zn	Mn	Fe	Cr	As	Pb
Cu	**1**	0.299	0.328	0.392	**0.773**	0.140	0.203	0.277
Ni	0.299	**1**	0.522	−0.595	0.198	0.341	−0.009	0.482
Zn	0.328	0.522	**1**	0.050	0.385	−0.058	−0.378	0.311
Mn	0.392	−0.595	0.050	**1**	0.365	−0.177	0.110	−0.218
Fe	**0.773**	0.198	0.385	0.365	**1**	−0.098	0.053	0.026
Cr	0.140	0.341	−0.058	−0.177	−0.098	**1**	**0.660**	0.239
As	0.203	−0.009	−0.378	0.110	0.053	**0.660**	**1**	−0.149
Pb	0.277	0.482	0.311	−0.218	0.026	0.239	−0.149	**1**

**Table 6:** Physical characteristics of Sabga smectic clay.

Parameters	Pore volume (cm^3^·g^−1^)	Micropores volume (cm^3^·g^−1^)	Specific surface area (m^2^·g^−1^)	Cation exchange capacity (CEC)	pH
SB01	0.2	0.04	90	42	5.5

**Table 7:** Composition in oxides of Sabga clay.

Sample	Elements											
	SiO_2_	Al_2_O_3_	Fe_2_O_3_	MnO	MgO	CaO	Na_2_O	K_2_O	TiO_2_	P_2_O_5_	L.O.I	Total
SB01	63.23	13.57	6.60	0.01	0.44	0.68	0.21	0.44	0.21	—	10.51	100.91

L.O.I: loss on ignition.

**Table 8:** Mineral composition (wt. %) of Sabga smectite.

Sample	Elements					
	Montmorillonite	Kaolinite	Cristobalite	Felspars	Ilmenite	Heulandite
SB01	59	—	38	8	1	—

**Table 9:** Results of the adsorption test by smectic clay: adsorbent mass: 0.5 g.

Heavy metals	Initial concentration (mg·L^−1^)	Residual concentration (mg·L^−1^)	Adsorption capacity (mg·g^−1^)	% Adsorption
Cu	0.01	0.004	0.6	60
Zn	0.2	0.022	17.8	89
Mn	0.04	0.018	2.2	55
Fe	3.90	7.40	—	−89.74
Pb	0.011	0	1.1	100

**Table 10:** Results of the adsorption test by smectic clay: adsorbent mass: 1 g.

Heavy metals	Initial concentration (mg·L^−1^)	Residual concentration (mg·L^−1^)	Adsorption capacity (mg·g^−1^)	% Adsorption
Cu	0.01	0.002	0.4	80
Zn	0.2	0.033	8.35	83.5
Mn	0.04	0.022	0.9	45
Fe	3.90	9.38	—	−140.51
Pb	0.011	0	0.55	100

**Table 11:** Comparison of adsorption capacity of manganese (II) ions with other adsorbents.

Absorbents	Adsorption capacity (mg·g^−1^)	References
Untreated volcanic ash	0.303	[47]
Kaolinite	0.004	[48]
Granular activated carbon	0.025	[47]
Sabga smectite	2.2	Present study

**Table 12:** Comparison of adsorption capacity of lead (II) ions with other adsorbents.

Absorbents	Adsorption capacity (mg·g^−1^)	References
Jordanian Kaolinite clay	1.84	[49]
Sabga smectite	1.1	Present study

**Table 13:** Comparison of the results with similar works: same and different points.

Works	Location	Physical analysis of water				Heavy metals analysis of water	Characterization of sediments	Water treatment
		pH	EC (*μ*s·cm^−1^)	Tu (NTU)	SS (mg·L^−1^)			
[8]	Galma Zaria, Nigeria	6.02–7.40	930–0120	—	9–81	No heavy metals analysis	Physical characterization of sediments	No water treatment
[9]	Hire, Ivory Coast	5.9–7.9	60–2660	—	—	As, Pb, Cd, Cr, Zn, and Hg pollution	No sediment characterization	No water treatment
[50]	Bibiani, Ghana	8.1–8.3	2160–2650	1.2–5.8	41–96	As and Fe pollution	No sediment characterization	No water treatment
[51]	Surow River, Ghana	6.57–7.69	0.20–0.95	30–850	—	Fe and Hg pollution	No sediment characterization	No water treatment
[3]	Betare-Oya, East Cameroun	5.42–7.58	11.60–122.10	2.30–4758	2.00–8996	Cu, Fe, Mn, As, and Pb pollution	No sediment characterization	No water treatment
[52]	Birim River, Ghana	6.36–7.02	58.03–128.9	31.66–3000	—	Fe pollution	Fe, Cd, and As concentrations are above the WHO limit	No water treatment
[53]	Tangandougou, Mali	No physical characterization				Cd pollution; no Pb and As pollution	No sediment characterization	No water treatment
[6]	Gorontalo, Indonesia	No physical characterization				As, Hg, and Pb pollution	As, Hg, and Pb pollution	No water treatment
[5]	Lower Pra Basin, Ghana	4.90–7.90	60–1140	—	—	Fe, Mn, Pb, Al, and Hg pollution	No sediment characterization	No water treatment
[10]	Djouzami, Adamawa Cameroon	6.59–7.02	23.3–24.9	38.23–253.3	46–318	Cd, Ni, Pb, and Fe pollution	No heavy metals pollution in sediments	Lead adsorption with Bana smectite 100% maximum removal after adding 0.5 g
Present study	Betare-Oya, East Cameroon	6.3–7.1	25.5–43	60–237.67	50.5–666.6	Mn, Fe, As, and Cd pollution	Cu, Ni, Mn, Fe, and Cr pollution	Cu, Zn, Mn, Fe, and Pb water treatment test

## Data Availability

No data were used to support this study.
